# Supplementary material for: Intrinsic mechanisms in the gating of resurgent Na+ currents
Source: eLife. 2022 Jan 25;11:e70173. doi: 10.7554/eLife.70173 (PMC8824471; doi:10.7554/eLife.70173)
Supplement: Transparent reporting form [file elife-70173-transrepform1.pdf]

Westbrook Centre
Milton Road
Cambridge CB4 1YG, UK

P 01223 855340
W elifesciences.org
T @elife

eLife's transparent reporting form

We encourage authors to provide detailed information *within their submission* to facilitate the interpretation and replication of experiments. Authors can upload supporting documentation to indicate the use of appropriate reporting guidelines for health-related research (see EQUATOR Network), life science research (see the BioSharing Information Resource), or the ARRIVE guidelines for reporting work involving animal research. Where applicable, authors should refer to any relevant reporting standards documents in this form.

If you have any questions, please consult our Journal Policies and/or contact us: editorial@elifesciences.org.

Sample-size estimation

- You should state whether an appropriate sample size was computed when the study was being designed
- You should state the statistical method of sample size computation and any required assumptions
- If no explicit power analysis was used, you should describe how you decided what sample (replicate) size (number) to use

Please outline where this information can be found within the submission (e.g., sections or figure legends), or explain why this information doesn't apply to your submission:

> The experiments here were designed to quantify the detailed time- and voltage-dependent properties of the Nav currents in cerebellar Purkinje neurons to facilitate the development of a computational model to recapitulate the gating of the underlying Nav channels. Sample sizes were determined based on mean data (and associated standard deviations) obtained from analyses of voltage-clamp recordings of Nav currents in mouse cerebellar Purkinje neurons acquired and described previously (Ransdell et al., 2017). This information is provided in the Methods section under 'Electrophysiological recordings and analysis' (lines 197-199).

Replicates

- You should report how often each experiment was performed
- You should include a definition of biological versus technical replication
- The data obtained should be provided and sufficient information should be provided to indicate the number of independent biological and/or technical replicates
- If you encountered any outliers, you should describe how these were handled
- Criteria for exclusion/inclusion of data should be clearly stated
- High-throughput sequence data should be uploaded before submission, with a private link for reviewers provided (these are available from both GEO and ArrayExpress)

Please outline where this information can be found within the submission (e.g., sections or figure legends), or explain why this information doesn't apply to your submission:

eLife Sciences Publications, Ltd is a limited liability non-profit non-stock corporation incorporated in the State of Delaware, USA, with company number 5030732, and is registered in the UK with company number FC030576 and branch number BR015634 at the address Westbrook Centre, Milton Road, Cambridge CB4 1YG.

The numbers of cells used to obtain each referenced experimental result are reported in the Results section and in the Figure legends. All of the experimental data presented in this manuscript reflect biological replicates. Technical replicates (repeating voltage-clamp paradigms on the same cell) were occasionally acquired to ensure that the properties of the Nav currents did not change during prolonged whole-cell recordings. Technical replicates are not included in the data presented. This information is provided in the Methods section under 'Electrophysiological recordings and analysis' (lines 199-204).

The criteria for the inclusion (or exclusion) of acquired voltage-clamp data are described in the Methods section (lines 206-207).

No outliers were encountered.

eLife Sciences Publications, Ltd is a limited liability non-profit non-stock corporation incorporated in the State of Delaware, USA, with company number 5030732, and is registered in the UK with company number FC030576 and branch number BR015634 at the address Westbrook Centre, Milton Road, Cambridge CB4 1YG.

Statistical reporting

- Statistical analysis methods should be described and justified
- Raw data should be presented in figures whenever informative to do so (typically when N per group is less than 10)
- For each experiment, you should identify the statistical tests used, exact values of N, definitions of center, methods of multiple test correction, and dispersion and precision measures (e.g., mean, median, SD, SEM, confidence intervals; and, for the major substantive results, a measure of effect size (e.g., Pearson's r, Cohen's d)
- Report exact p-values wherever possible alongside the summary statistics and 95% confidence intervals. These should be reported for all key questions and not only when the p-value is less than 0.05.

Please outline where this information can be found within the submission (e.g., sections or figure legends), or explain why this information doesn't apply to your submission:

> Means and standard error of the means are reported in the Results section and in the Figure legends. The statistical test used in the analyses of the data presented in Figure 5 is indicated in the Figure legend; the exact p-value obtained is provided in the legend to Figure 5. The approach used for the numerical optimization of the computational model is described in the Methods section (lines 228-236).

(For large datasets, or papers with a very large number of statistical tests, you may upload a single table file with tests, Ns, etc., with reference to sections in the manuscript.)

Group allocation

- Indicate how samples were allocated into experimental groups (in the case of clinical studies, please specify allocation to treatment method); if randomization was used, please also state if restricted randomization was applied
- Indicate if masking was used during group allocation, data collection and/or data analysis

Please outline where this information can be found within the submission (e.g., sections or figure legends), or explain why this information doesn't apply to your submission:

> In this manuscript, we have compared acquired experimental data with results obtained in computational simulations; experimental group allocation was not necessary.

Additional data files ("source data")

- We encourage you to upload relevant additional data files, such as numerical data that are represented as a graph in a figure, or as a summary table
- Where provided, these should be in the most useful format, and they can be uploaded as "Source data" files linked to a main figure or table
- Include model definition files including the full list of parameters used
- Include code used for data analysis (e.g., R, MatLab)
- Avoid stating that data files are "available upon request"

Please indicate the figures or tables for which source data files have been provided:

> Model definition files and Matlab scripts used for experimental simulations have been uploaded to https://github.com/morenomdphd/Resurgent_INa. This link is provided in the Methods section (lines 219-220).

eLife Sciences Publications, Ltd is a limited liability non-profit non-stock corporation incorporated in the State of Delaware, USA, with company number 5030732, and is registered in the UK with company number FC030576 and branch number BR015634 at the address Westbrook Centre, Milton Road, Cambridge CB4 1YG.
